# Bigger Is Not Always Better: Effects of Electrocautery Setting on Tissue Injury in a Porcine Model

**DOI:** 10.7759/cureus.26841

**Published:** 2022-07-14

**Authors:** Austin L Shiver, Colton Webber, Taylor Sliker, Patrick Rushford, Aaron Shaw

**Affiliations:** 1 Orthopaedics, Augusta University Medical College of Georgia, Augusta , USA; 2 Orthopaedics, Augusta University Medical College of Georgia, Augusta, USA; 3 Pathology and Laboratory Medicine, Augusta University Medical College of Georgia, Augusta, USA; 4 Orthopaedic Surgery, Dwight D. Eisenower Army Medical Center, Fort Gordon, USA; 5 Orthopaedic Surgery, Dwight D. Eisenhower Army Medical Center, Fort Gordon, USA

**Keywords:** basic orthopedic sciences, ­wound healing, orthopaedic surgery, bovie, electrocautery

## Abstract

Introduction

Electrosurgery for dissection and hemostasis remains one of the foundational tools for the field of surgery as a whole. Monopolar cautery remains the most utilized modality for achieving the aforementioned goals. Given the prolonged history and pre-modern development of "Bovie" cautery, there remains a paucity of data regarding appropriate settings and intensity for various tissue types, procedures, or locales. As a result, utilized settings depend on precedent and personal preference. We aimed to determine the amount of secondary soft tissue injury by volume and depth beyond the electrocautery pen tip in the skin and subcutaneous tissue as well as skeletal muscle.

Methods

Porcine samples were used for experimental testing using two testing types: 1) skin and subcutaneous tissue and 2) Skeletal muscle. Sample sizes were standardized at 1 cm3 cubes. For skin samples, tissue injury was created with either a scalpel or electrocautery pen on cut setting, and tested at intensities from 10 to 150 in increments of 10. Skeletal muscle samples were similarly tested using the electrocautery pen only in either a cut or coagulation setting. Samples were tested at incremental intensities from 10 to 120 for both settings. Electrocautery was tested for a period of five seconds with a continuous current. All samples were placed in formalin and underwent histologic staining with hematoxylin and eosin staining to be assessed for the extent of tissue injury in terms of depth, radius, and volume. The measurements were recorded in millimeters.

Results

For skin incision, there was a positive and significant correlation with respect to the radius (R=.73, p=0.006). When considering intensity with an interval of 10-70 there was a positive and significant correlation with respect to the radius, depth, and volume. The cold knife incision had no notable soft tissue injury beyond the depth of the incision. Regarding skeletal muscle, again, a significant and positive correlation between increasing monopolar settings was noted for both the coagulation and cut functions (R=.84, p=.0005; R=0.84, p=0.0006). A positive correlation was found between increasing cut intensity and volume of soft tissue injury (R=0.73, p=.008); this was not reflected in the coagulation setting. When limited to an intensity range of 10-60, a significant relationship was noted for depth, radius and volume (R=.95, p= <0.001; R=0.98, p= <.001; R=.92, p=.001).

Conclusion

In all samples, apart from the cold knife skin incision, additional soft tissue injury beyond the tip of the electrocautery pen was noted. Given our findings, recommendations include using the lowest setting required for the purposes of the given surgical case as well as minimizing electrocautery use for skin incisions given its association with a larger volume of tissue injury in comparison with a scalpel. Additionally, electrocautery should be used with care in, and around neurovascular structures as soft tissue injury did occur several millimeters beyond the tip of the electrocautery pen. Further study is needed to see if these patterns are similar in living animals as well as human tissue and whether they bear any clinical impact on surgical wound healing or other surgical complications.

## Introduction

Soft tissue management is an important aspect of any surgical procedure. Different instruments and techniques have been utilized to minimize blood loss and expedite operative time, chief among these tools is monopolar electrocautery. Given the fact that current passes through tissues adjacent to the surgical site to the grounding pad elsewhere on the patient, it is not unreasonable to assume that there may be soft tissue injury beyond the tip of the electrocautery pen. Similarly, there remains incompletely elucidated interaction and potential injury at the site of surgery. While electrocautery achieves the goals of decreasing surgical times and blood loss, how it is applied varies widely between specialties and even providers within the same specialty in terms of settings and intensities. Currently, there is a lack of any recommendations regarding what setting or intensity to use for a given location or procedure. The purpose of the study is to determine if the amount of soft tissue injury beyond the tip of the electrocautery pen is proportional to the settings utilized. 

## Materials and methods

Skin

Samples of fresh never frozen porcine skin and subcuticular fat of the size 2 cm x 2cm x 1cm were prepared with a butcher knife. A Covidien© brand was used to simulate intra-operative electrocautery. A 1 cm incision was made with either a #10 blade scalpel or an electrocautery pen on the “cut” setting in the center of the sample. The sample was then placed immediately in formalin. Overall, 17 samples were obtained, one using a cold knife and 16 using the cut function on the electrocautery pen with intensity settings starting at 10 and increasing to 150 at intervals of 10, and one sample at the maximum setting of 300 for the machine. 

Skeletal muscle

Two cm^3^ samples of fresh never frozen porcine skeletal muscle were obtained commercially and prepared with a carving knife. A Covidien© brand was used to simulate intra-operative electrocautery. The tip of the electrocautery pen was placed at the center of the porcine skeletal muscle cube and pressed just into the surface of the cube. The electrocautery pen was fired for a timed five-second burst. The porcine skeletal muscle sample was then placed immediately in formalin. Overall, 24 samples were obtained using the cut and coagulation functions on the electrocautery pen with intensity settings starting at 10 and increasing to 120 at intervals of 10. The maximum intensity setting for the coagulation function was 120 and was chosen at the intensity cap for both the coagulation and cut settings. 

Histological preparation

Each sample was bisected at the cautery site. Both bisected tissue pieces were placed in separate labelled histology cassettes for further fixation and paraffin embedding. The tissue blocks were cut for slides and stained via hematoxylin and eosin. The lateral and vertical depth of tissue damage on each slide was measured via a 10x ocular micrometer and 10x objective lens. The measurements were recorded in millimeters. 

Statistical analysis

In order to determine an approximate volume of soft tissue injury, the histologic samples appeared to best approximate a cone shape. Therefore, the following equation was utilized: Volume: 1/3πr2h where π: 3.14159, r: the widest lateral extent of injury, and h: depth of injury. Measurements for depth, radius, and volume were made for both coagulation and cut modalities. These were then assessed with a paired T-test as calculated by as well as for a correlation with Pearson's product-moment correlation coefficient. Statistical significance was assigned for a p-value of < 0.05.

## Results

Skin

With respect to skin incision, there was no predictable relationship between the settings of the cut function of the monopolar electrocautery pen and the depth (R = -.48) of soft tissue injury (Table 1). There was a positive and significant correlation with respect to the radius (R=.73, p=.006) and volume (R=.60, p =.03 ) (Table 1). When considering the intensity interval of 10-70, there was a significant and positive correlation between increasing electrocautery settings and radius (R=.82, p =.001), depth (R=.85, p=.0003), and volume (R= .82, p=.001) (Table 1). When compared with the cut function samples, the cold knife incision had no notable soft tissue injury beyond the depth of the incision and had no skin charring at the surface of the skin which was subjectively increased with higher settings on the cut function.

**Table 1:** Skin secondary injury depth, radius, and volume for "cut" function R value - Pearson's Product-Moment Correlation Coefficient; T stat - Paired T-test

Intensity Setting (watts)	Depth of Tissue Damage (mm)	Radius of Tissue Damage (mm)	Volume of Tissue Damage (mm^3^)
10	5.00	2.45	31.43
20	4.20	2.80	34.48
30	6.50	2.30	36.01
40	5.70	2.90	50.19
50	7.90	2.80	64.86
60	6.80	3.80	102.83
70	8.30	5.20	235.02
80	6.55	1.75	21.01
90	3.75	5.65	125.36
100	3.00	6.60	136.85
110	4.00	6.25	163.62
120	3.50	6.90	174.50
130	3.00	7.05	156.14
140	3.70	8.40	273.39
150	6.00	5.65	200.57
300	3.00	7.30	167.41
R Value	-0.4881	0.7354	0.6011
T-Stat	-1.768	3.431	2.378
P-value	0.1074	0.0064	0.0387

Skeletal muscle

There was a significant and positive correlation between increasing electrocautery settings and depth of injury on both the cut and coagulation settings (R=.84, R=.84, and p =.0005, p = .0006) (Table 2). There was no relationship noted between increasing intensity settings and radius for cut (R=.36, p=.24) or coagulation (R =-.0019, p = .99) (Table 2). However, a positive and significant correlation was found between increasing cut intensity settings and volume of soft tissue injury (R=.72, p = .008) that was not observed with the coagulation setting (R = .27 and p=.38) (Table 2). There was a significant and positive correlation noted for the coagulation setting in depth (R: .95, p = < .001), radius (R: .98, p = <.001), and volume (R= .92, p = .001) for the intensity interval of 10-60 (Table 3). There was no correlation noted for the cut setting in this same interval for depth (R=.59), radius (R=.35), and volume (R=.54) (Table 2). 

**Table 2:** Skeletal muscle secondary injury depth, radius, and volume for both "coagulation" and "cut" functions R value - Pearson's Product-Moment Correlation Coefficient; T stat - Paired T-test

Intensity Setting (watts)	Coagulation Depth (mm)	Cut depth (mm)	Coagulation Radius (mm)	Cut Radius (mm)	Coagulation Volume (mm^3^)	Cut Volume (mm^3^)
10	0.50	0.55	2.40	1.50	3.01	1.29
20	2.10	0.75	3.00	3.60	19.79	10.17
30	2.10	0.85	5.15	3.10	58.32	8.55
40	2.60	1.50	5.20	3.55	73.62	19.79
50	3.00	2.10	7.00	2.35	153.93	12.14
60	3.80	0.90	8.45	3.25	284.13	9.95
70	2.40	2.25	8.00	3.67	160.84	31.82
80	2.00	3.00	5.95	7.00	74.14	153.93
90	4.40	7.00	7.80	1.52	280.33	17.04
100	8.80	7.60	2.50	3.50	57.59	97.49
110	7.90	7.00	3.00	3.70	74.45	100.35
120	7.60	4.50	3.20	4.55	81.49	97.55
R value	0.848	0.8407	-0.0019	0.363	0.2763	0.7214
T-Stat	5.060	4.911	0.006	1.232	0.9091	3.294
P-value	0.0004	0.0006	0.9952	0.246	0.3846	0.008

**Table 3:** Skeletal muscle secondary injury depth, radius and volume for "coagulation" and "cut" functions for intensity 10-60 R value - Pearson's Product-Moment Correlation Coefficient; T stat - Paired T-test

Intensity Setting (watts)	Coagulation Depth (mm)	Cut Depth (mm)	Coagulation Radius (mm)	Cut Radius (mm)	Coagulation Volume (mm^3^)	Cut Volume (mm^3^)
10	0.50	0.55	2.40	1.50	3.01	1.29
20	2.10	0.75	3.00	3.60	19.79	10.17
30	2.10	0.85	5.15	3.10	58.32	8.55
40	2.60	1.50	5.20	3.55	73.62	19.79
50	3.00	2.10	7.00	2.35	153.93	12.14
60	3.80	0.90	8.45	3.25	284.13	9.95
R value	0.9506	0.5935	0.9824	0.3565	0.9285	0.5416
T-stat	9.686	2.332	16.63	1.206	7.912	2.037
P-value	2.126	0.0418	0.0001	0.2552	1.295	0.0689

## Discussion

Electrocautery was first described clinically by Cushing in his landmark paper in 1928, having used it on October 1, 1918, on cranial vascular myeloma [1]. Clinical applications for electrosurgery had previously been explored by multiple physicians utilizing fulguration and desiccation [2]. It became readily apparent that a new era of surgical intervention had begun as this modality afforded an ability for soft tissue dissection with improved hemostasis. As noted by O’Conner, “Bovie's machine permitted surgery with minimal blood loss, low infection rate, and little tissue damage [2].” It is estimated that 80% of all cutting and coagulation performed today are accomplished with electrosurgical instruments [3]. Along with improved hemostasis, and likely a result of such hemostasis, the surgical time has also decreased with the advent of modern electrosurgery. However, given its advent prior to the modern era, rigorous pre-clinical testing/evaluation is lacking and there remains a wide variability in its clinical use regarding settings utilized as well as settings in relation to specific body location. Electrosurgical modalities have been evaluated by gastrointestinal endoscopists, general surgeons, otolaryngologist, and in the field of gynecology [4-8]. There remains wide variability between specialties as well as intra-specialty variability regarding settings with use often predicated on tradition/precedent. Monopolar electrocautery requires current to travel through tissues to the dispersal electrode which draws current back to the production unit and as such comes with a specific risk profile. Complications of monopolar electrocautery have been described to include burns, intraoperative fire, interference with implanted medical devices, and injury secondary to insulation failure and coupling, both direct and capacitive [6,8]. Townsend noted an injury secondary to unsuspected energy transfer occurring between 0.6 and 5 per 1000 operations [9]. Liu noted remote burns in the setting of gynecologic laparoscopy [6]. Sankaranarayanan et al. noted 40,000 patient burns annually and over 600 million USD in claims for those injuries. “Care should be taken when operative electrosurgical devices, particularly monopolar devices. The sparking effect at the tool tip may cause an explosion when it encounters inflammable gases that are often used for anesthesia during the operation. Further, the current traveling through the body can interfere with any implanted devices such as pacemakers and defibillators [3].” Townsend et al. noted in 2015 a potential for remote burns at the site of neuromonitoring lead secondary to antenna type coupling [10]. Remote histologic injury to the femoral artery was noted in an animal model after monopolar use in a simulated lumbar spine surgery dissection by Bayram [11]. Further, Chang et al point out that “while prized for hemostatic control and dissection capability, conventional electrosurgical devices are associated with significant thermal damage to incised tissues, low surgical precision with the potential for injury to adjacent structures (e.g. bowel, nerves, blood vessels), and delayed wound healing [4].”

 Descriptions of monopolar cautery and associated tissue injury are relatively sparse in general although there are descriptions in various gynecologic, oral medicine and general surgical/endoscopic procedures [4-7, 12-16]. These studies have evaluated histologic findings related to monopolar cautery of human myometrium and tonsillar tissue, murine fascia, porcine epidermis, skeletal muscle, gallbladder, vaginal tissue, and rabbit muscle/liver. These studies have variously defined the zone of injury associated with monopolar use, employed various histologic methodologies for evaluation, and have variable/non-sequential utilization of power settings. Bowers evaluated rabbit muscle with MRI and histology at 24h and 14d utilizing monopolar electrocautery in both coagulation and cut modalities as well as ferromagnetic loop and noted worst healing in the MPE-coag group [12]. Moreover, Chang et al noted worse healing at 6 weeks in murine fascia when MPE was used for both cut and coagulation modes compared to cold knife and pulsed radiofrequency [4]. Ultrasonic scalpel was evaluated against MPE by Homayoufar et al. in a porcine model and they noted a similar quality of necrosis between the two groups but significantly more in the ultrasonic scalpel group [17]. 

Given the paucity of data/descriptions in skeletal muscle and orthopaedic literature in general as it relates to monopolar electrocautery at various setting levels, we set out to evaluate the amount of local soft tissue injury beyond the tip of the “bovie” pen. Further, we determined to evaluate if the injury is proportional to the settings utilized for skin, subcutaneous tissue, and skeletal muscle. 

**Figure 1:**
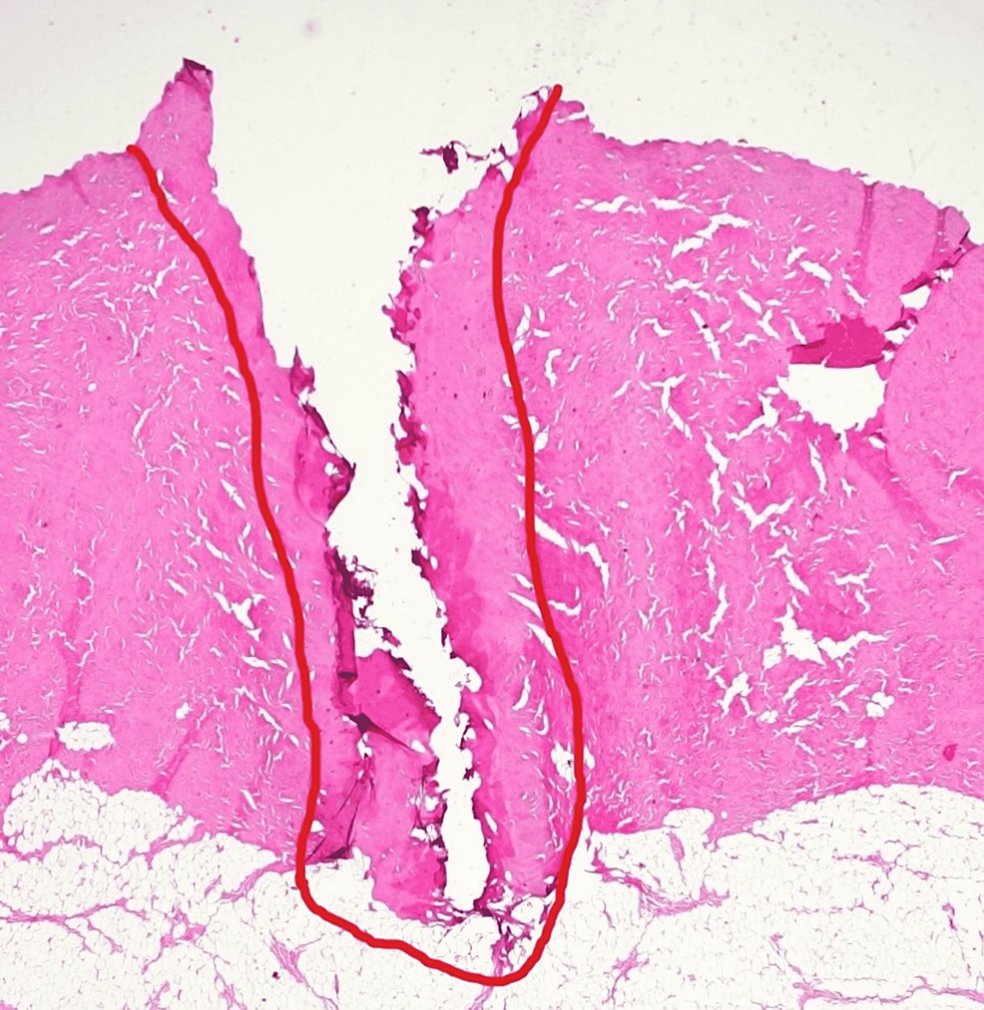
Skin/Subcutaneous tissue at intensity 10 on cut function Representative histologic image of skin/subcutaneous tissue with zone of secondary injury outlined

**Figure 2:**
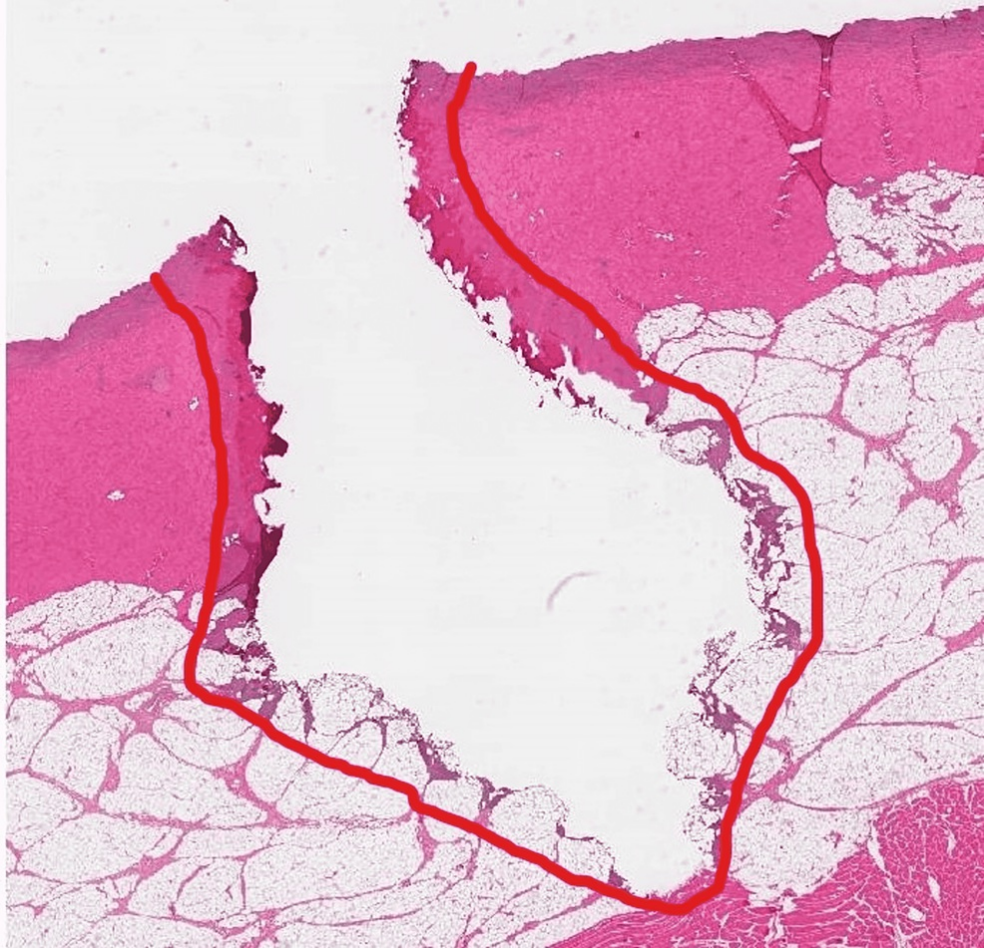
Skin/Subcutaneous tissue at intensity 70 on cut function Representative histologic image of skin/subcutaneous tissue with zone of secondary injury outlined

With respect to skin incision, there were significant differences between the cold knife sample and the “cut” sample. Grossly, there was progressively worsening charring of the skin with electrocautery in comparison with the scalpel incision. One could surmise improved local hemostasis through the skin and subcutaneous tissue layers in exchange for potential problematic wound healing as well as cosmetic issues with increasing intensities of cut electrocautery during skin incision. However, there is limited data regarding this effect in living human models. Histologically, there was no correlation positively or negatively between increasing “cut” setting intensity and depth or volume of soft tissue injury. There was a weakly positive but significant correlation between radius and volume of soft tissue injury. An interesting finding is that when the intensity interval of 10-70 is considered in isolation, is that a significant and positive correlation is observed for depth, radius, and volume. This suggests a more consistent and predictable arc of current through the soft tissues that breaks down and becomes unpredictable at higher intensities. Representative images outline the zone of injury in this range of intensities (Figures 1, 2).

**Figure 3:**
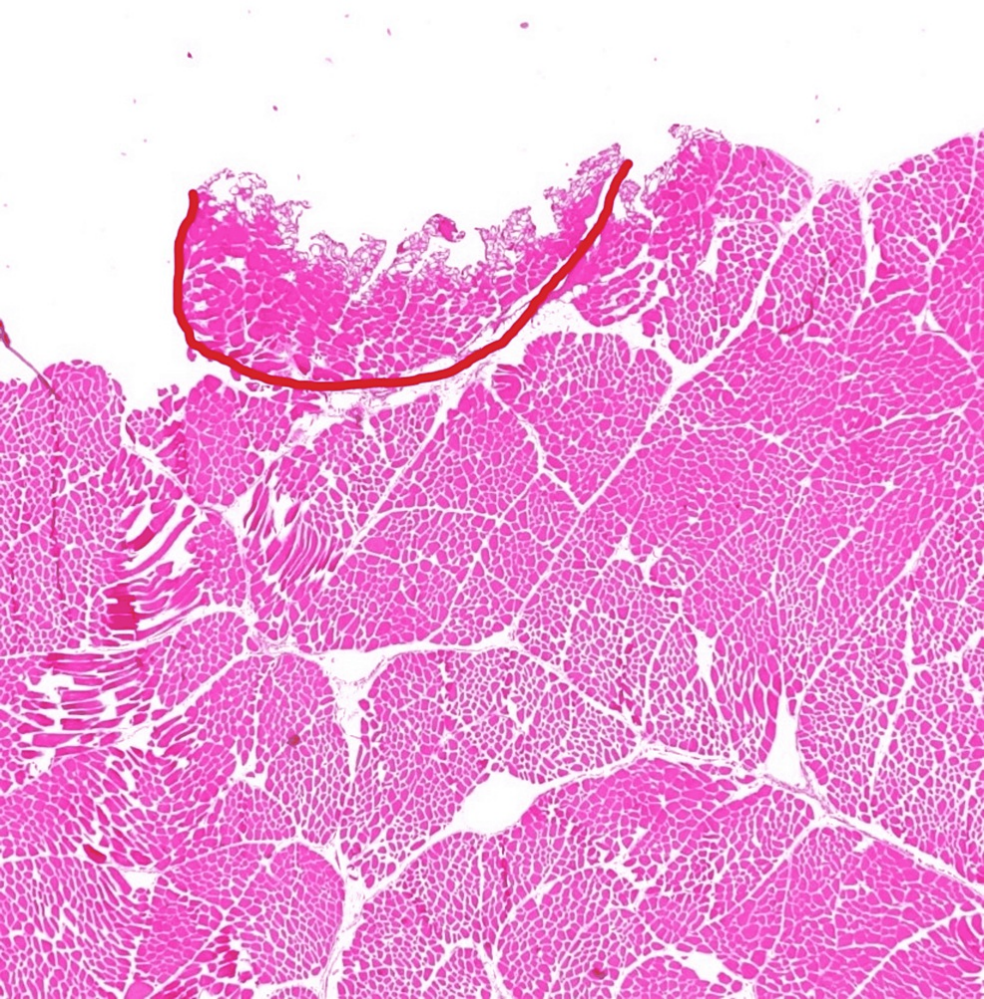
Skeletal muscle tissue at intensity 10 on coagulation function Representative histologic image of skeletal muscle with zone of secondary injury outlined

**Figure 4:**
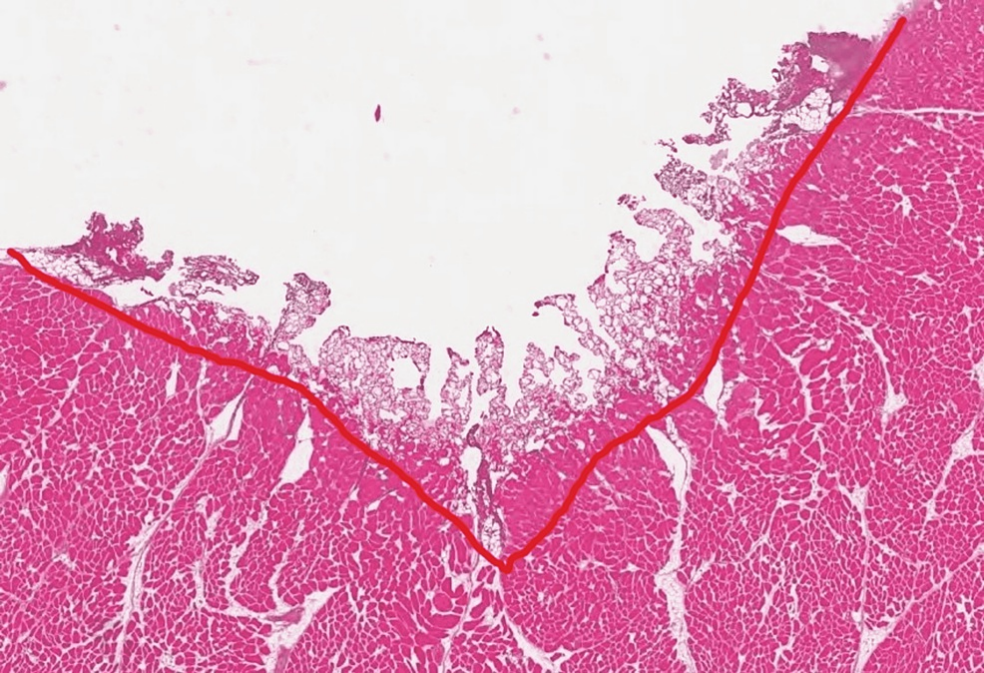
Skeletal muscle tissue at intensity 60 on coagulation function Representative histologic image of skeletal muscle with zone of secondary injury outlined

**Figure 5:**
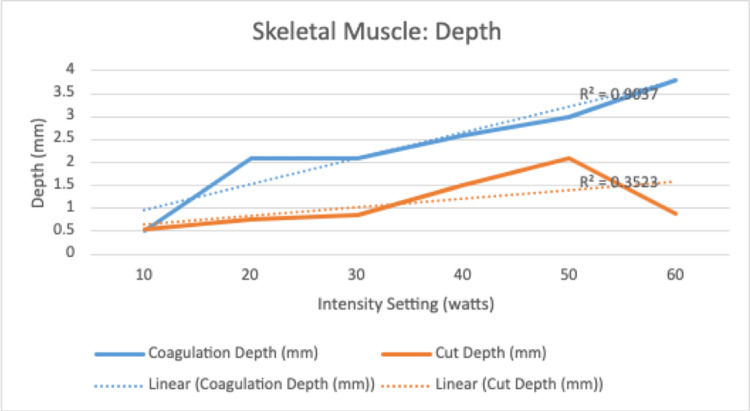
Linear correlation for secondary injury for coagulation and cut functions at intensity 10-60

**Figure 6:**
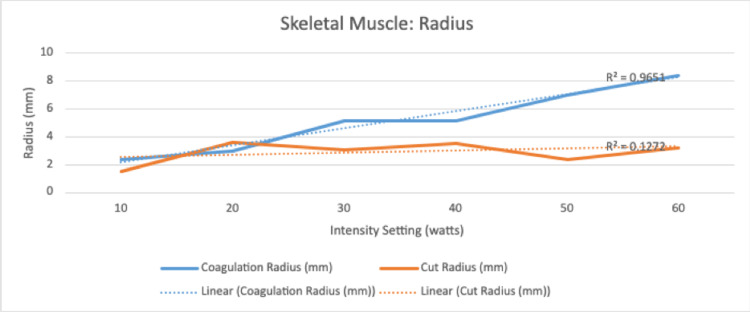
Linear correlation for secondary injury for coagulation and cut functions at intensity 10-60

**Figure 7:**
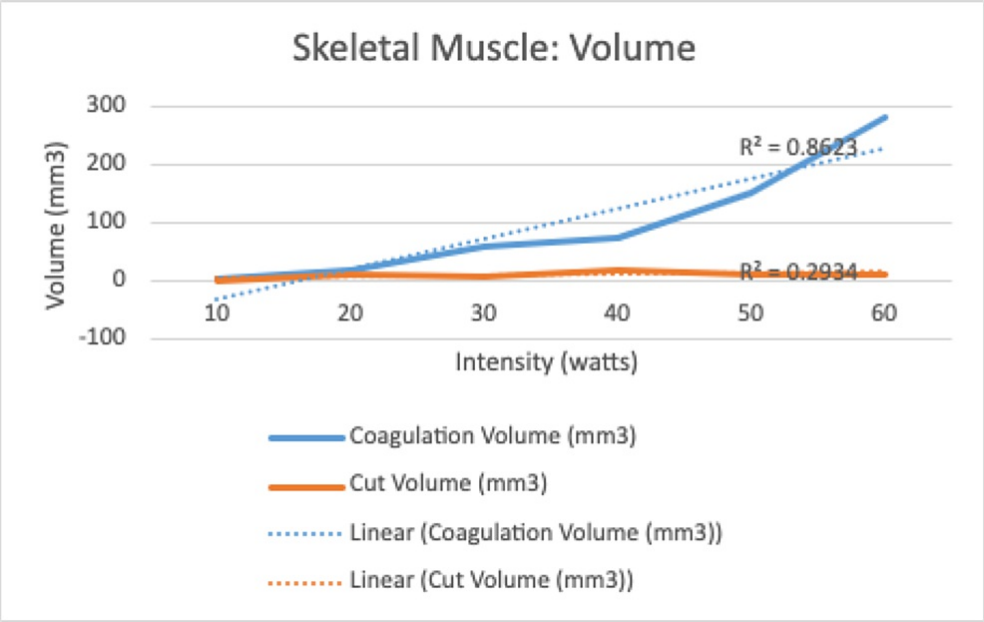
Linear correlation for secondary injury for coagulation and cut functions at intensity 10-60

Regarding skeletal muscle, there was no significant relationship between the increasing intensity of “cut” or “coagulation” setting and radius of soft tissue injury. There was a weakly positive but significant correlation between the increasing intensity of the “cut” setting and the volume of soft tissue injury. This was not reciprocated for the coagulation setting. However, there was a significant positive correlation for depth of tissue injury for both settings. This is clinically important with respect to surgical dissection particularly around neurovascular bundles as many orthopaedic surgical exposures require dissection near or exposure of these bundles where careful attention to surrounding anatomy is paramount. Histologically, this is represented in Figures 3 and 4. Interestingly, when evaluated in the intensity interval of 10-60, a strongly positive and significant correlation for depth, radius, and volume of soft tissue injury for the “coagulation” setting only was noted (Figures 5-7). This was not observed for the “cut” setting and the previously weakly positive correlation for the volume of soft tissue injury was lost for this interval. As previously mentioned, this would suggest a reliable and predictable current arc through skeletal muscles on the “coagulation” setting at these intensity levels. This also suggests the inverse regarding the “cut” setting in skeletal muscle yields an unpredictable soft tissue injury, perhaps due to an inconsistent current arc through the tissues. These trends demonstrated 

Limitations

This study has several notable limitations. First, this is a non-living porcine model. As such, variability and differences between porcine and human tissue may be possible. Again, as the tissue utilized was in non-living specimens healing response was not evaluated. Additionally, histologic measurements were made by a single pathologist while effective at removing inter-rater bias does introduce intra-rater bias. Also, this study was performed using a single electrocautery system and its findings may not be generalizable to other machines. Finally, the application of the monopolar implement was done manually which would allow for variation in depth and pressure applied. 

## Conclusions

The choice of dissection tool should be one of the active decisions. Based on these findings, we would recommend skin incision be made by scalpel exclusively as there is no additional soft tissue injury at the level of the skin. If the electrocautery is to be utilized for skin incisions, we would recommend the lowest possible intensity on the “cut” setting, but to avoid intensities above 70 on the Covidien© electrocautery system, as the surrounding soft tissue injury becomes unpredictable. When considering the utilization of the MPE pen one should remain aware of the structures adjacent to the tip of the instrument the structures deep to the tip of the electrocautery pen. We would also recommend avoiding intensities beyond 60 Covidien© electrocautery system for the “coagulation” as the tissue injury beyond that setting was significantly unpredictable. If amenable to the situation, knife or scissor dissection should be considered given its relative precision with less surrounding soft tissue injury. Further, we advocate an active consideration of all available electrosurgical instrument options prior to surgical intervention. 
